# Effectiveness of Chitosan versus Natural Aloe Vera on Candida Adherence in Denture Soft Lining Material

**DOI:** 10.1155/2024/9918914

**Published:** 2024-01-08

**Authors:** Muhammad Rizwan Memon, Hina Memon, Mehwish Shoro, Humera Bhurgri, Rakhi Issrani, Azhar Iqbal, Osama Khattak, Mosa Altassan, Afaf A. Almabadi, Sherif El Sayed Sultan, Hussein Abdelfatth Ismail, Muhammad Nadeem Baig, Namdeo Prabhu

**Affiliations:** ^1^Department of Prosthetic Dental Sciences, College of Dentistry, Jouf University, Sakaka, Saudi Arabia; ^2^Department of Prosthodontics, Institute of Dentistry, Liaquat University of Medical and Health Sciences, Jamshoro, Pakistan; ^3^Department of Preventive Dentistry, College of Dentistry, Jouf University, Sakaka, Saudi Arabia; ^4^Department of Research Analytics, Saveetha Dental College and Hospitals, Saveetha Institute of Medical and Technical Sciences, Saveetha University, Chennai, India; ^5^Department of Restorative Dentistry, College of Dentistry, Jouf University, Sakaka, Saudi Arabia; ^6^Department of Oral and Maxillofacial Prosthodontics, Faculty of Dentistry, King AbdulAziz University, Jeddah, Saudi Arabia; ^7^Department of Fixed Prosthodontics, College of Dentistry, Tanta University, Tanta, Egypt; ^8^Department of Prosthodontics, Zagazig University, Zagazig, Egypt; ^9^Department of Oral and Maxillofacial Surgery and Diagnostic Sciences, College of Dentistry, Jouf University, Sakaka, Saudi Arabia

## Abstract

**Background:**

Soft denture lining materials act as a cushion between the denture base and tissues. Alongside having many advantages, its main problem is candida growth due to its rubbery and porous texture. Many interventions have been performed to halt the growth of candida within soft lining materials such as the use of antifungal therapy and strict oral and denture hygiene but there are consequences such as recurrence, drug resistance, and toxicity related to these interventions. Since natural agents such as aloe vera and chitosan have been proven to have antibacterial and antifungal properties with minimum adverse effects, this study aimed to study the effectiveness of chitosan and aloe vera powders incorporated within denture soft lining materials against candida adherence. *Methodology*. A total of 60 soft-lining material samples were prepared that were equally divided into three groups, viz., group 1 (chitosan incorporation), group 2 (aloe vera incorporation), and group 3 (control). Candida was obtained from the microbiology lab to form a candidal suspension, diluted in 0.9% NaCl to match the McFarland standard bacteriologic solution. Samples were incubated at 37°C for 24 hours in test tubes containing 100 mL of the candidal suspension and 9.9 mL of the previously prepared Sabouraud dextrose agar. Crystal violet stain was used to stain the adhering cells by fixing them with methanol 80%. For each sample, the adhering candida cells were counted on three standard fields by using an inverted light microscope, and the mean of those fields was recorded.

**Results:**

The mean value for samples containing aloe vera was 41.15, while the mean values for samples containing chitosan and the control group were 16.05 and 79.1, respectively. Of all the three groups, aloe vera powder had a significant efficacy against candida growth as compared to the chitosan and control groups (*P* value = 0.001).

**Conclusion:**

Both herbal agents were effective against candida growth. In comparison, aloe vera was more effective against candida growth compared to chitosan.

## 1. Introduction

Certain problems such as alveolar bone resorption, sharp ridges, or a friable nature of supporting mucosa result in an uneven distribution of stresses to denture-supporting tissues, leading to tissue injury, sore spots, patient discomfort, and altered fit of prosthesis [1, 2]. To overcome these problems, soft denture lining materials can be used. Soft denture lining materials are made up of resilient materials that are applied over the denture-bearing surface of the prosthesis. These materials serve as a cushion, absorbing the masticatory load and its traumatic effects on the tissues supporting the dentures [3, 4].

Resilient denture liners are made of silicone elastomers or plasticized acrylic resins. They may be used for both short- and long-term purposes, and they could be heat- or auto-polymerized [5, 6]. In order to provide denture wearers with the greatest possible benefits, the ideal soft lining material should have a variety of characteristics, such as low water solubility, resistance to microbial growth, good resiliency, biocompatibility, and dimensional stability [7, 8]. Despite this, there are a variety of challenges with soft lining materials, including their failure to bond to denture bases, loss of resilience, color changes, poor tear strength, porosity, and subsequent plaque accumulation with dominant *Candida albicans* (*C. albicans*) colonization [9]. This is one of the major issues that affect the soft lining material's long-term efficacy [10]. *C. albicans* is a common yeast found in the oral cavity that can adhere to dentures, host cells, bacteria, and other candida cells. This adhesion leads to the creation of biofilm, which makes the organisms resistant to antimicrobial and antifungal medicines [11–13].

Denture-induced stomatitis is the most common clinical illness caused by *C. albicans*, a fungus that is also responsible for causing certain other oral infections within the oral cavity [10]. It is frequently accompanied by poor oral hygiene, poor diet, smoking, trauma from wearing dentures continuously, decreased salivary flow, or inadequate denture base material or quality [10].

Denture stomatitis can be challenging to manage because of its complex aetiology. There have been many different treatment methods suggested such as maintaining oral and denture hygiene, removing dentures at night time, using topical or systemic antifungal medications, diet modification, and relining or replacing prosthetics [14, 15]. Antifungal therapy for denture stomatitis may offer symptomatic relief, but the recolonization of fungi within the oral cavity cannot be prevented, which ultimately causes its recurrence. Moreover, it is also accompanied by adverse consequences such as drug-resistant fungus and the toxicity of currently available medications. Additionally, elderly people, who make up a greater proportion of the denture-wearer population, are unable to take the recommended dosage of the medication due to poor motor skills [14–16].

There is a global interest in medicinal plant extracts since herbal therapy is thought to be a very reliable and safe alternative to antimicrobial medications with few to no side effects. In order to prevent the colonization of candida, care has been taken to modify the soft denture base lining material through the addition of herbal and natural agents such as chitosan, aloe vera, and mint oil [10, 17].

Aloe vera is considered as the most popular plant species used, both medically and economically [18]. More than 200 different biologically active compounds, including amino acids, anthraquinones, enzymes, hormones, minerals, salicylic acid, saponins, steroids, carbohydrates, and vitamins, have been identified in the plant based on its chemistry. Its biological functions include the ability to heal wounds, acting as an antibacterial agent, reducing inflammation, acting as an antioxidant, acting as allergen suppressant, and moisturizing the skin [19–22].

In addition, aloe vera has been proven to have potent antibacterial properties that are useful in the treatment of gingival diseases. It also reduces soft tissue edema, which in turn decreases gingival bleeding. Along with its potent antibacterial properties, which are beneficial in treating periodontal pockets where standard cleaning is challenging, it contains antifungal characteristics that may aid in the treatment of denture stomatitis. Its antiviral qualities are also documented to aid in the treatment of cold sores (herpes simplex) and shingles (herpes zoster) [22].

On the other hand, chitosan, a natural polymer derived from crustacean outer shells, is suited for use in several medicinal applications due to its antifungal and antibacterial properties. It was suggested as a bioadhesive to the oral mucosa since it is nontoxic [23]. Its oligomers interfere with the growth of fungal cells by interacting with their growth-promoting enzymes and diffusing them into hyphae [24, 25].

Considering the effectiveness of both natural agents on candida growth, this study aimed to compare the antifungal effectiveness of chitosan and aloe vera powders on mean reduction in fungal growth incorporated in heat polymerised soft denture lining material, since as per the author's knowledge, none of the studies had been conducted before to study this parameter in comparison.

## 2. Materials and Methods

This *in vitro* experimental study was conducted at the Prosthodontics Laboratory of the Institute of Dentistry, LUMHS, Jamshoro, Pakistan, and antifungal activity was tested at Medical Research Centre, LUMHS, Jamshoro, Pakistan, with a total sample size of 60, divided into three groups: group 1 (chitosan incorporation), group 2 (aloe vera incorporation), and group 3 (control) by using a nonprobability convenience sampling technique. Only standard dimension, freshly fabricated and sterilized heat-cured soft denture lining plates (6 × 6 × 2 mm), and freshly incubated *C. albicans* species were included in this study.

### 2.1. Mould Preparation

**Figure 1:**
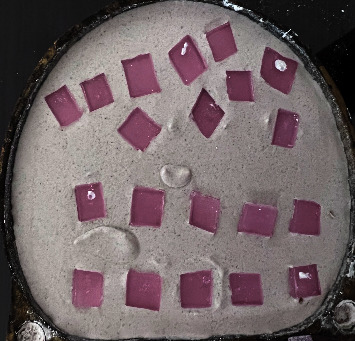
Mould fabrication by using modelling wax placed in freshly mixed dental stone.

After taking approval from the Ethical Review Committee of LUMHS, Pakistan, a total of 60 multiple specimens of modelling wax (Kemdent Tenatex Eco, UK) were fabricated in dimensions of 6 × 6 × 2 mm and placed in freshly mixed dental stone (ISI HI-TECH; according to manufacturer's instructions W/P ratio: 32 mL/100 g) within the lower portion of the dental flask as shown in Figure 1. When the dental stone was completely set, a separating media was applied to the dental stone and allowed to dry. The upper part of the flask was completely filled with the dental stone and kept until set. The flask was opened afterwards and wax patterns were removed leaving a space for the soft liner pattern [22].

### 2.2. Specimen Preparation

#### 2.2.1. Proportioning, Mixing, and Fabrication of Soft Liner/Chitosan Powder Specimen

As recommended by the manufacturer, a mixing ratio of 1.2 g per 1 mL was utilized with the soft liner (GC Tokyo, Japan). In order to obtain an exact P/L ratio, the weight of the chitosan powder (Sigma Company; 2% of the total wt. of powder component) was removed from the total weight of the soft liner powder during the mixing process [3]. A clean glass jar with a lid was used for mixing. When the soft lining material reached the dough stage, it was taken out by the hands, placed on the flask that had been previously prepared, and covered with a polyethylene sheet. To ensure that the soft lining material was distributed evenly inside the mould and to remove any excess material, the upper portion of the flask was placed on it, and it was subjected to a hydraulic pressure of 100 kg/cm^2^ for 5 minutes. The flask was removed from the press and opened and the extra material and polyethylene were scraped away by using a wax knife. The packed flask was once more kept under pressure for 5 minutes, after which it underwent a 90-minute cure at 70°C following the manufacturer's instructions, followed by a 30-minute temperature increase to 100°C. After the curing cycle was complete, the flask was removed from the water bath and left to cool for 30 minutes at room temperature before keeping them under tap water for a further 15 minutes. Specimens were retrieved and excess material was cut down with sharp scalpel blades followed by finishing with fine-grit silicone polishing burs and fine-grit sandpaper [26, 27].

#### 2.2.2. Proportioning, Mixing, and Fabrication of Soft Liner/Aloe Vera Powder Specimen

As recommended by the manufacturer, a mixing ratio of 1.2 g per 1 mL was utilized with the soft liner (GC Tokyo, Japan). In order to obtain an exact P/L ratio, the weight of the aloe vera powder (aloe vera powder prepared after drying fresh aloe vera leaves plucked from a plant in sunlight after 15 days) was removed from the total weight of the soft liner powder during the mixing process [3]. A clean glass jar with a lid was used for mixing. When the soft lining material reached the dough stage, it was taken out by the hands, placed on the flask that had been previously prepared, and covered with a polyethylene sheet. To ensure that the soft lining material was distributed evenly inside the mould and to remove any excess material, the upper portion of the flask was placed on it, and it was subjected to a hydraulic pressure of 100 kg/cm^2^ for 5 minutes. The flask was taken out of the press and opened, and the extra material and polyethylene were scraped away by using a wax knife. The packed flask was once more kept under pressure for a further five minutes, after which it underwent a 90-minute cure at 70°C following the manufacturer's instructions, followed by a 30-minute temperature increase to 100°C. After the curing cycle was complete, the flask was taken out of the water bath and left to cool for 30 minutes at room temperature before keeping them under tap water for a further 15 minutes. Specimens were retrieved and excess material was cut down with sharp scalpel blades followed by finishing with fine-grit silicone polishing burs and fine-grit sandpaper [26, 27].

### 2.3. Isolation of Candida

In order to create a candidal suspension with 10 CFU/mL of 0.5 McFarland standards, yeast was obtained from a microbiology lab and diluted in 0.9% NaCl. This solution matches the McFarland standard bacteriologic solution.

Sabouraud dextrose agar was made, autoclaved for 15 minutes at 121°C/15 pressure, and stored in accordance with the manufacturer's instructions. All soft lining samples were placed into the tubes with 100 mL of the candidal suspension and 9.9 mL of previously prepared Sabouraud dextrose agar. Following this, the tubes were incubated at 37°C for 24 hours.

**Figure 2:**
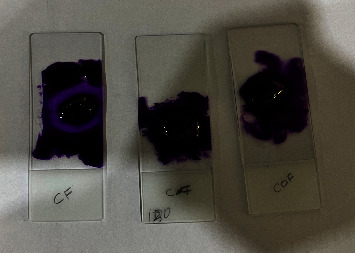
Staining of adherent candida cells with crystal violet.

**Figure 3:**
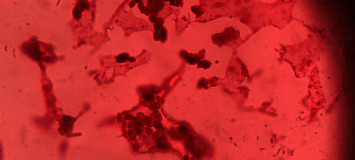
Candida cells as seen under an inverted light microscope.

All samples were taken out after incubation and rinsed five times in autoclaved deionized water to eliminate any loosely attached cells. Crystal violet stain was used to stain the adhering cells for 1 minute after fixing them with methanol 80% for 30 seconds as shown in Figure 2 [28]. For each sample, the adhering candida cells were counted on three standard fields by using an inverted light microscope, and the mean of those fields was recorded as shown in Figure 3. Filamentous forms were not counted in order to standardize the measurement of adhering cells, whereas budding daughter cells were counted as separate yeast [29].

### 2.4. Statistical Analysis

Data were analyzed using the Statistical Package for the Social Sciences version 20.0 (IBM Corp, Armonk, NY, USA). Aloe vera- and chitosan-infused denture soft liners' mean values were compared. By using the one-way ANOVA test, a comparison of the means of groups was made. A *P* value of ≤0.05 was considered significant.

## 3. Results

By examining the stained specimens for evaluating the adherence ability of *C. albicans* to soft liners for each group under the inverted light microscope, the mean values obtained for the control, chitosan, and aloe vera groups were 79.1, 41.15, and 16.05. Of all the three groups, it was found that the aloe vera powder had a significant efficacy against candida growth as compared to the chitosan and control groups (*P* value = 0.001) as shown in Table 1.

## 4. Discussion

The viscoelastic characteristics of soft liners enable them to act as a cushion between the denture and the edentulous ridge by evenly distributing the occlusal forces over the denture-bearing area but an increased risk of candidal infection has been noted with the use of soft lining materials [22]. Natural antimicrobial compounds have been recommended as an alternative to synthetic, systemic, or local antibiotics due to their antibacterial and antifungal properties, as well as their availability and affordability [30, 31]. In numerous studies, the addition of various antifungal drugs and nanoparticles in the soft denture liner material has been attempted to prevent the colonization of fungus [10, 32].

Growing interest has been observed in chitosan modification and application in the biomedical field because of its biocompatibility, biodegradability, nontoxic characteristics, and antibacterial activity [33, 34]. On the other hand, aloe vera is renowned for its significant therapeutic benefits and is one of the most beneficial plants in nature for health. This could be a result of the presence of a new protein with a molecular weight of 14 kDa that has antifungal and anti-inflammatory capabilities. This protein works by preventing trypsin from performing its protease-inhibitory activity [19]. In the current study, chitosan and aloe vera powders were added to soft liners in an effort to utilize their antifungal characteristics. According to the findings of this study, the number of *C. albicans* cells adhering to the surface of the soft lining material containing chitosan and aloe vera powders has significantly decreased when compared to the control specimen group.

Saeed et al. incorporated two different types of chitosan into tissue conditioners, i.e., tissue conditioner modified by chitosan and tissue conditioner modified by chitosan oligosaccharide and compared them with the control group. They found that compared to the control group, experimental groups showed more reduction in the number of colony-forming units of *C. albicans*. Additionally, the tissue conditioner modified by chitosan oligosaccharide, once immersed in saliva, exhibited improved inhibition until the third day as compared to the tissue conditioner modified by chitosan [35]. Mohammaed and Fatalla investigated the antifungal effects of 1.5 wt% and 2 wt% of chitosan nanoparticles on heat-cured acrylic-based soft lining material and found a highly significant decrease in the number of candida cells adhered to the soft liner after incorporating 1.5 wt% and 2wt% of chitosan compared to samples of the control group [22]. According to Abdulwahhab and Jassim, adding aloe vera powder (3% and 10%) to heat cure acrylic soft lining material powder causes a statistically significant decrease in *C. albicans*' cell count in comparison to the control group. Additionally, improvement in shear bond strength and tear strength was also noted [3]. A recent study by Nair et al. showed that aloe vera had the least antifungal activity when compared to the conventional denture cleansers, neem and triphala. In the current study, while comparing the mean reduction in candida count between the three groups, aloe vera was shown to be more effective than chitosan and the control group [36].

We were unable to compare our findings with the previous research since no such studies have explored the effectiveness of chitosan powder versus aloe vera powder used in the denture soft lining against the adherence of candida. Further research is required to determine the effect of chitosan and aloe vera powders on the mechanical properties of denture soft liners since both natural substances have been reported to be beneficial against the growth of candida. Additionally, when naturally dried aloe vera powder particles were mixed with soft lining material in the current investigation, there was a mild brownish discolouration of the material. Therefore, it is proposed that additional research is needed to assess the impact of commercially available and fresh aloe vera gels in order to address material discolouration.

### 4.1. Limitations

Our primary aim was not only to evaluate the antifungal efficacy of natural substances but also to examine the influence of these substances on the mechanical properties of soft denture liners. Unfortunately, due to certain limitations imposed by permissible restrictions, we were unable to utilize the resources and equipment available at a different institution to investigate the mechanical effects. Accordingly, it is recommended that another study should be undertaken to assess the influence of incorporating these materials on the mechanical properties of soft liners. Furthermore, it is essential to explore the antifungal properties of these materials when utilizing various resin types for the fabrication of complete dentures. A notable limitation of this study was the scarcity of data available in the literature, which prevented us from conducting a comparative analysis with other studies. Therefore, it is recommended that similar investigations should be undertaken in other regions. Additionally, further research is needed to evaluate the antifungal efficacy of dried aloe vera powder in comparison to the clear gel found in aloe vera leaves. Such findings will facilitate our recommendation of aloe vera as a reliable antifungal agent.

## 5. Conclusion

From the results of this research, we can conclude that both chitosan and aloe vera powders can be regarded as strong antifungal materials and their incorporation within the soft denture lining material can help achieve the antifungal activity against candida microorganisms. Furthermore, aloe vera powder was found to be more effective than chitosan. The findings of this study recommend the need to further evaluate the effects of these natural agents on the mechanical properties of denture soft liner materials.

## Figures and Tables

**Table 1:** Comparisons for adherent candida cells under the microscope for different groups.

Sample no.	Chitosan	Aloe vera	Control	*F*	*P* value
Sample 01	47	18	89	270.569	0.001
Sample 02	27	07	73		
Sample 03	30	07	61		
Sample 04	35	18	59		
Sample 05	30	26	94		
Sample 06	45	23	94		
Sample 07	45	08	88		
Sample 08	53	12	82		
Sample 09	45	15	79		
Sample 10	39	10	78		
Sample 11	47	13	75		
Sample 12	56	25	83		
Sample 13	46	17	88		
Sample 14	33	23	57		
Sample 15	31	09	83		
Sample 16	43	17	84		
Sample 17	38	17	77		
Sample 18	51	23	91		
Sample 19	43	15	78		
Sample 20	39	18	69		

Mean ± SD	41.15 ± 8.19	16.05 ± 5.98	79.1 ± 10.97	—	—

Between groups (mean square)	20151.7			—	—

Within groups (mean square)	74.5			—	—

## Data Availability

The dataset used in the current study will be made available on request from Dr. Muhammad Rizwan Memon, dr.muhammad.rizwan@jodent.org, mrmemon@ju.edu.sa.
